# The cosmopolitan maternal heritage of the Thoroughbred racehorse breed shows a significant contribution from British and Irish native mares

**DOI:** 10.1098/rsbl.2010.0800

**Published:** 2010-10-06

**Authors:** M. A. Bower, M. G. Campana, M. Whitten, C. J. Edwards, H. Jones, E. Barrett, R. Cassidy, R. E. R. Nisbet, E. W. Hill, C. J. Howe, M. Binns

**Affiliations:** 1 McDonald Institute for Archaeological Research, University of Cambridge, Cambridge, UK; 2 Department of Archaeology, University of Cambridge, Cambridge, UK; 3 Department of Biochemistry, University of Cambridge, Cambridge, UK; 4 Department of Veterinary Basic Sciences, Royal Veterinary College, London, UK; 5 Research Laboratory for Archaeology and the History of Art, University of Oxford, Oxford, UK; 6 National Institute of Agricultural Botany, Cambridge, UK; 7 Department of Anthropology, Goldsmiths College, London, UK; 8 Sansom Institute for Health Research, University of South Australia, Adelaide, South Australia, Australia; 9 School of Agriculture, Food Science and Veterinary Medicine, University College Dublin, Republic of Ireland

**Keywords:** Thoroughbred horse, foundation of breed, maternal origins, phylogenetics, mitochondrial DNA

## Abstract

The paternal origins of Thoroughbred racehorses trace back to a handful of Middle Eastern stallions, imported to the British Isles during the seventeenth century. Yet, few details of the foundation mares were recorded, in many cases not even their names (several different maternal lineages trace back to ‘A Royal Mare’). This has fuelled intense speculation over their origins. We examined mitochondrial DNA from 1929 horses to determine the origin of Thoroughbred foundation mares. There is no evidence to support exclusive Arab maternal origins as some historical records have suggested, or a significant importation of Oriental mares (the term used in historic records to refer to Middle East and western Asian breeds including Arab, Akhal-Teke, Barb and Caspian). Instead, we show that Thoroughbred foundation mares had a cosmopolitan European heritage with a far greater contribution from British and Irish Native mares than previously recognized.

## 1. Introduction

The English Thoroughbred is the best known breed of horse in the western world. Thoroughbreds were developed during the seventeenth and eighteenth centuries in England, largely owing to the enthusiasm of English aristocracy for horse racing and betting [1]. The paternal origins of the breed are well documented as being derived from a handful of Middle Eastern stallions, the most influential of which are *Godolphin Arabian*, *Darley Arabian* and *Byerley Turk* [2]. Yet, the origins of Thoroughbred mares are less well known. The General Studbook (GSB), the breed registry for Thoroughbred horses, first published in 1791 [3], documents Thoroughbred pedigrees back to seventeenth century foundation bloodstock, identifying 74 foundation mares. Present day membership of the GSB requires comprehensive records, including genetic verification of parentage. However, in the early history of the breed, only minimal details of founding mares were recorded, as females were not regarded as important [4]. Since then, the contribution of mares to race performance has been acknowledged [5,6], but the origins of female Thoroughbred lineages are contentious, with a history of intense speculation [7–9]. This speculation is primarily focused on the contribution of Arab and/or ‘Oriental’ mares (the term used in historic records to refer to Middle East and western Asian breeds including Arab, Akhal-Teke, Barb and Caspian [9]).

Maternally inherited mitochondrial DNA (mtDNA) has been used for tracing maternal bloodlines in Thoroughbreds [6,10] and to study geographical origins of domestic horses [11–13]. Using mtDNA, we tested four hypotheses for the origins of Thoroughbred maternal lineages: Thoroughbred foundation mares were (i) imported Arabs [7], (ii) Oriental [9], i.e. imported from the Middle East and western Asia; (iii) native to the British Isles [8], and (iv) mares from a variety of origins depending on availability at the time and place.

## 2. Material and methods

Whole-genomic DNA was extracted from horse hair roots according to standard protocols. Polymerase chain reactions were set up as previously published [12]. We obtained 247 base pairs of mitochondrial D-loop from 196 Thoroughbred horses and 83 British Native horses (Fell, *n* = 16; Highland, *n* = 24; Shetland, *n* = 43). Sequences were deposited in GenBank (www.ncbi.nlm.nih.gov): Thoroughbred: EU580148–EU580172; Fell: GU563629–GU563645; Highland: GU563646–GU563668; Shetland: GU563669–GU563712.

Our data were compared with 1550 horse D-loop sequences available from GenBank (www.ncbi.nlm.nih.gov). Breeds represented by fewer than 10 individuals were not included in analyses. Together, the data represented 30 major Thoroughbred maternal lineages ([10]; 296 Thoroughbreds), 201 Oriental horses (Arab, Akhal-Teke, Barb and Caspian) and 255 British Native and Irish horses (Connemara, Exmoor, Fell, Irish Draught, Kerry Bog and Shire) and horse breeds from across Eurasia (table 1; for details of breed and sample number see electronic supplementary material, table S1). Horses were grouped by geographical population: British Isles (*n* = 255), Central Asia (*n* = 38), China and the Far East (*n* = 339), Eastern Europe (*n* = 39), Lowlands and Central Europe (*n* = 153), Mediterranean (*n* = 435), Middle East and western Asia (*n* = 201), the North and Russia (*n* = 72), Scandinavia (*n* = 25) and Siberia and Mongolia (*n* = 76) (for details see electronic supplementary material, tables S2 and S3).

**Table 1.** The proportion of clades within populations of domestic horses. (Haplotype definitions are after Jansen *et al*. [11]. Clade C is partitioned into two, named C1 and C2, for consistency with published literature since there is no phylogenetic basis for their amalgamation into a single clade as previously reported [1].)

proportion of clades per population	*n*	A (%)	B (%)	C1 (%)	C2 (%)	D (%)	E (%)	F (%)	G (%)	H (%)
Thoroughbred horses	296	11	13	6	14	55	2	0	0	0
British and Irish Native horses	255	17	8	17	8	31	14	4	0	0
Arab horses	91	43	20	5	0	18	0	14	0	0
Oriental horses (excluding Arabs)	110	36	11	5	1	26	3	17	1	0
European horses	670	32	7	5	8	33	0	10	4	0
Central and East Asian horses	507	37	6	6	2	19	3	23	2	1
total horses	1929	29	8	7	6	31	4	13	2	0

Genetic groups (haplotypes) were defined using median-joining networks drawn according to Lei *et al*. [13]. These handle large datasets effectively, allow for multi-state data [14] and are commonly used for within-species comparisons where sequence variation is limited [15,16]. Population statistics were calculated and AMOVA [17] performed using Arlequin v. 3.11 [18]. Correspondence analyses (CA) were conducted using Adegenet [19]. Neighbour-joining trees were constructed in MEGA v. 4.1 [20]. Mixed stock analysis was performed using SPAM v. 3.7 [21]. SPAM v. 3.7 implements a conditional maximum-likelihood approach to estimate contributions of donor populations (Arab, Oriental and British Natives) to mixed populations (Thoroughbreds).

**Figure 1.**
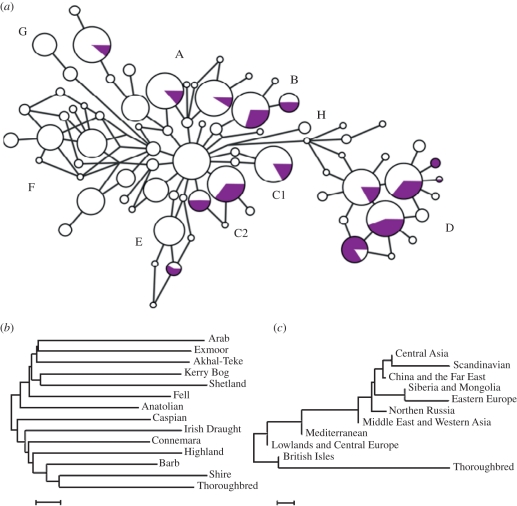
(*a*) Median-joining network of 1929 mitochondrial D-loop sequences from domestic horses, and (*b*) neighbour-joining trees based on mean pairwise differences between breeds (scale bar, 0.002) and (*c*) geographical regions (scale bar, 0.05), including Thoroughbred (purple circles in (*a*)), British and Irish Native, Arab, Oriental breeds and published sequences from domestic horses from European, Middle Eastern, Asian and Far Eastern populations. Nodes in the network are proportional to the frequency of haplotypes. Haplotypes are defined following Jansen *et al*. [11].

The nomenclature of Jansen *et al*. [11] was used to define haplotypes within networks (figure 1*a*). Jansen *et al*. define haplotypes C1 and C2 as a single clade, however, there is no phylogenetic basis for this. For consistency with published literature, we retained Clade C nomenclature, but present Clades C1 and C2 separately. Associations among haplotype frequencies within and between populations were investigated using correspondence analysis and Fisher's exact tests [22].

## 3. Results

Thoroughbreds showed extensive haplotype sharing with Eurasian domestic horses (figure 1*a*), with the exclusion of Clades F, G and H and the ancestral Clade A6 ([11]; table 1). AMOVA partitioned 90 per cent of total genetic variation among individuals within Thoroughbreds. Therefore, Thoroughbred mares encompass the majority of genetic variation within Eurasian horse populations. These data are consistent with a history of genetic amalgamation, rather than an origin from a single distinct population.

**Figure 2.**
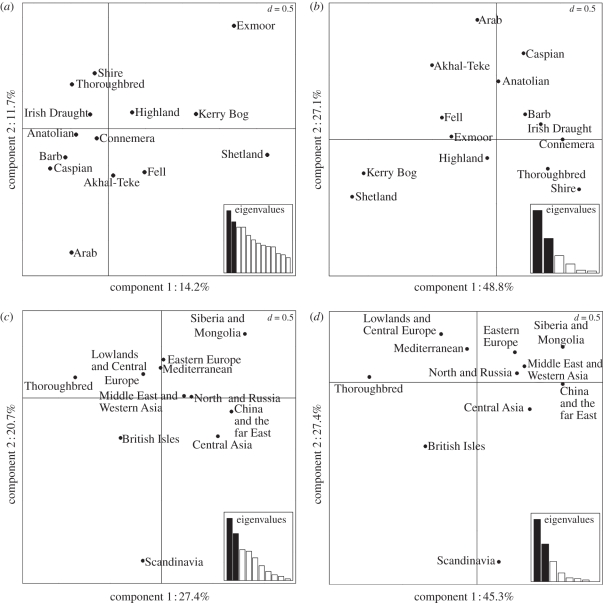
Correspondence analyses by (*a*,*c*) allele and (*b*,*d*) haplogroup frequencies. Associations within and between breeds (*a*,*b*) show that Thoroughbred horses have closer affinity to British Native than to Oriental horses (Arab, Barb, Turkmen, Akhal-Teke and Caspian). Associations within and between geographical groupings (*c*,*d*) show that Thoroughbred horses have closer affinity to British Native and European horses than Middle East and western Asian (including Arab and Oriental) horses. *D*-values denote scale of grid; scree plots indicate relative importance of plotted components.

Using CA of allele frequencies (figure 2*a*) and haplotype frequencies (figure 2*b*), we compared Thoroughbreds with British and Irish Native and Oriental horse breeds (including Arabs) to determine the origins of Thoroughbred foundation mares. CA confirmed the separation of Thoroughbred horses from Arab horses (figure 2*a*,*b*), with *χ*^2^ distances between Arabs and the average population being greater than that between Thoroughbreds and the average population. Pairwise genetic distances (table 2) showed that Thoroughbreds had closest affinity to Connemara (*F*_ST_ = 0.004) and Irish Draft horses (*F*_ST_ = 0.016) and were distantly related to Arab horses (*F*_ST_ = 0.177) compared with other breeds. This indicates that Thoroughbreds had a cosmopolitan rather than pure-Arabian origin. Furthermore, CA showed that Thoroughbreds had greater affinity to British and Irish Native breeds than Oriental horses, with the exception of Barbs. Pairwise genetic distances (table 2) showed that Thoroughbreds were distantly related to Exmoor horses (*F*_ST_ *=* 0.267), indicating that British Native breeds were not used indiscriminately.

**Table 2.** Pairwise genetic distances (*F*_ST_) between Thoroughbred, Arab, Oriental and British Native horses. (Negative values result from the inaccuracy of the Arlequin v. 3.11 algorithm's estimates when *F*_ST_ values are near zero, especially when combined with the small sample sizes of the Anatolian, Caspian, Connemara and Shire breeds.)

	sample size	Arab	Barb	Connemara	Exmoor	Fell	Highland	Irish Draught	Kerry Bog	Shetland	Shire	Anatolian	Akhal-Teke	Caspian	Thoroughbred
Arab	91	0													
Barb	39	0.14563	0												
Connemara	12	0.08677	−0.00380	0											
Exmoor	20	0.09055	0.30030	0.19895	0										
Fell	17	0.05112	0.14297	0.04770	0.05444	0									
Highland	31	0.14676	0.03789	−0.00859	0.21283	0.07692	0								
Irish Draught	59	0.10999	0.01385	−0.03144	0.19569	0.07238	−0.00329	0							
Kerry Bog	39	0.09330	0.18727	0.09537	0.07111	0.00600	0.10611	0.11335	0						
Shetland	67	0.08771	0.10768	0.06122	0.13153	0.03722	0.07534	0.08729	0.02866	0					
Shire	10	0.21392	0.02757	0.03068	0.36180	0.21061	0.03676	0.02552	0.24863	0.16964	0				
Anatolian	15	0.03507	0.08765	−0.02486	0.12612	−0.00406	0.04349	0.01830	0.05165	0.05968	0.14503	0			
Akhal-Teke	43	0.01365	0.14128	0.05945	0.06990	0.00430	0.09887	0.07940	0.03026	0.04344	0.19541	0.00305	0		
Caspian	13	0.07485	0.07342	−0.02968	0.16090	0.03059	0.00446	−0.01136	0.07179	0.07854	0.11638	−0.01169	0.03322	0	
Thoroughbred	296	0.17748	0.03192	0.00485	0.26696	0.14049	0.02494	0.01645	0.18103	0.14579	−0.01855	0.07162	0.14716	0.05663	0

CA of geographical populations (figure 2*c*,*d*) show that Thoroughbreds have greater affinity to British Isles and European horses than Middle East and western Asian populations, i.e. Oriental horses. Multiple iterations of neighbour-joining trees based on mean pairwise differences between breeds or geographical regions (figure 1*b*,*c*) consistently placed Thoroughbreds with British and Irish Native horses.

Based on our data, Thoroughbred foundation mares were not exclusively Arab or Oriental. Rather, Thoroughbred mares were of cosmopolitan European origin, with contribution from Barbs and with British and Irish Native horses playing a greater part in the founding of the Thoroughbred breed than previously recognized. This is supported by the analysis of haplotype sharing. For example, Clade F is strongly associated with Middle East, west Asian and Far Eastern horses, including Oriental breeds (Fisher's exact test: *p* < 0.00001), yet no Thoroughbred horse sequence lies within Clade F (figure 1). If horses of an Oriental origin made a major contribution to the Thoroughbred, we would expect to find Clade F among the Thoroughbred sequences, if only at low frequency.

To estimate the proportion of contribution of Arab, Oriental and British Native horses to Thoroughbred horses, we performed mixed-stock analysis of allele frequencies, using SPAM v. 3.7 [21]. The estimated contribution of British and Irish Native horses was 61 per cent, whereas that of Arabs was 8 per cent. Oriental horses (without Arabs) contributed 31 per cent.

## 4. Discussion

Our data demonstrate that Thoroughbred foundation mares were of cosmopolitan European heritage, with contributions from British and Irish Native and Oriental horses. The contribution from British and Irish Native horses is close to twice that of Oriental horses. This British Native maternal influence, is apparent in the current Thoroughbred population, e.g. 2009 Kentucky Derby winner, *Mine That Bird*, probably has British Native maternal origins, since his founding matriarch, *Piping Peg's Dam*, foaled in 1690, is Clade C1 based on the haplotype of her direct female descendents (Clade C1 is strongly associated with British Native breeds: Fisher's exact test *p* < 0.000001).

Additional foundation mares came from European horse populations, although we cannot determine precisely which. Our data show a contribution from Barb mares. However, Barb horses have undergone extensive crossbreeding with European horses, including Iberian breeds [23]. Thoroughbred affinity to Barbs may, therefore, reflect this crossbreeding rather than an original contribution. The majority of Thoroughbreds belong to Clade D (55%), previously reported as being associated with Iberian horses [24]. Yet, Clade D is frequent among European horse populations (31%) and thus, we cannot delineate a contribution to Thoroughbred foundation mares from Iberian breeds as opposed to one from European horse breeds as a whole.

By contrast, Oriental mares made a limited contribution to Thoroughbred maternal lineages with a minimal contribution from Arabs. Thoroughbred foundation mares, therefore, most likely represent a cross-section of female bloodstock available at each stud participating in the foundation of the breed. While influential Thoroughbred breeders may still claim Thoroughbreds as purely Oriental (specifically Arab), our results argue strongly against this claim.
